# Lithocholic Acid Restores Gut Microbiota and Bile Acid Homeostasis to Improve Type 2 Diabetes

**DOI:** 10.3390/nu18020341

**Published:** 2026-01-21

**Authors:** Han Ge, Mengxiao Guo, Xin Chen, Lu Chen, Xin Yang, Dingzuo Ge, Liqiang Guo, Yue Luo, Guangbo Ge, Lei Zhang, Ruirui Wang

**Affiliations:** 1 Shanghai Innovation Center of Traditional Chinese Medicine Health Service, Shanghai University of Traditional Chinese Medicine, Shanghai 201203, China; 22023571@shutcm.edu.cn (H.G.); gmxdoctor@163.com (M.G.); chenxin110595@163.com (X.C.); gedingzuo@163.com (D.G.); 15135452771@163.com (L.G.); 22024646@shutcm.edu.cn (Y.L.); 2 State Key Laboratory of Integration and Innovation of Classic Formula and Modern Chinese Medicine, Shanghai University of Traditional Chinese Medicine, Shanghai 201203, China; 3 State Key Laboratory of Discovery and Utilization of Functional Components in Traditional Chinese Medicine, Shanghai Frontiers Science Center of TCM Chemical Biology, Institute of Interdisciplinary Integrative Medicine Research, Shanghai University of Traditional Chinese Medicine, Shanghai 201203, China; chenlu970707@163.com; 4 Section of Endocrinology, Internal Medicine, School of Medicine, Yale University, New Haven, CT 06510, USA; yang.xin@yale.edu

**Keywords:** lithocholic acid (LCA), gut microbiota, bile acids, *A. muciniphila*, type 2 diabetes mellitus

## Abstract

**Background:** Bile acids participate in several metabolic processes, and disturbances in their circulating profiles are commonly observed in type 2 diabetes. In a cohort of older adults, individuals with diabetes exhibited markedly lower concentrations of metabolites derived from lithocholic acid. These findings prompted further evaluation of the metabolic effects of lithocholic acid. **Methods:** We assessed the actions of lithocholic acid in a mouse model of diabetes induced by a high-fat diet and streptozotocin. Fasting glucose, insulin levels, lipid parameters, and measures of insulin resistance were evaluated. Gut microbial composition, short-chain fatty acids, fecal enzyme activities, intestinal barrier markers, and bile acid patterns were analyzed. In vitro assays examined the direct effects of lithocholic acid on *A. muciniphila* and bile acid metabolism. **Results:** Lithocholic acid supplementation lowered fasting glucose and insulin levels and improved insulin resistance. It shifted the gut microbial community toward a healthier structure, increased the abundance of *A. muciniphila*, and raised short-chain fatty acid concentrations. Fecal bile salt hydrolase and β-glucuronidase activity declined, and intestinal barrier markers improved. Lithocholic acid enhanced TGR5 expression and reduced FXR signaling in the ileum. In vitro, physiologically relevant concentrations promoted *A. muciniphila* growth and altered microbial bile acid metabolism. **Conclusions:** Lithocholic acid influences the interactions among gut microbes, bile acid pathways, and host metabolic regulation. These findings suggest that this compound may have value as a dietary component that supports metabolic health in type 2 diabetes.

## 1. Introduction

T2DM is a chronic metabolic disorder commonly associated with obesity and persistent inflammation [1]. Apart from genetic and dietary effects, the gut microbiota has been recognized as an important regulator of glucose and lipid metabolism [2]. Recent studies have shown that metformin can increase the abundance of beneficial gut microbes, including *A. muciniphila*, which is linked to glycemic control and intestinal barrier function [3]. These findings highlight the microbiota–host interactions as a potential target in T2DM management.

Among metabolic pathways mediated by gut microbiota, the bile acid (BA) metabolic axis has gained increasing attention. The liver synthesizes primary bile acids, which are then converted into secondary bile acids by microbial enzymes in the colon. This bidirectional “microbiota–BA axis” not only facilitates lipid absorption but also regulates host metabolism through bile acid receptors. In patients with type 2 diabetes, changes in the composition of the gut microbiota disrupt the conversion of primary bile acids into secondary bile acids, resulting in an excess of conjugated bile acids. This aggravates insulin resistance and inflammatory responses [4]. Conversely, bile acids (BAs) influence microbial ecology through their antimicrobial effects. Restoring the composition of BAs has been shown to improve metabolic function by reducing inflammation and restoring barrier function [5].

The metabolic actions of BAs are primarily regulated through the farnesoid X receptor (FXR) and Takeda G protein-coupled receptor 5 (TGR5). FXR plays a key role in regulating bile acid synthesis and intestinal homeostasis. However, excessive activation of FXR can inhibit the secretion of GLP-1, disrupt intestinal integrity and promote lipid accumulation [6]. In contrast, TGR5 activation improves glucose tolerance, stimulates incretin release and reduces inflammation [7]. Recent studies have proposed a dual-target strategy involving the inhibition of intestinal FXR and the activation of TGR5. This can be mediated by gut microbial activities, including the production of FXR antagonists such as TβMCA [8].

Lithocholic acid (LCA), a secondary BA derived from microbial 7α-dehydroxylation, serves as a strong Tgr5 agonist [9,10]. Recent studies suggest that LCA levels decrease in T1D, potentially due to reduced microbial synthesis. Interestingly, a higher abundance of LCAs correlates with better preserved β-cells, indicating their potential benefit in modulating the disease [11]. Furthermore, LCA and its metabolites have anti-inflammatory effects and enhance gut barrier integrity. They also protect against colitis and metabolic inflammation [12].

The diet- and low-dose streptozotocin-induced mouse model was chosen because it reflects key metabolic features of type 2 diabetes, including diet-related insulin resistance and disturbed glucose control, and thus allows in vivo assessment of metabolic regulation. The aim of this study was to examine the effects of LCA on glucose metabolism and gut microbiota–bile acid interactions in this model. To address this objective, we evaluated the metabolic effects of LCA using animal models and complementary in vitro anaerobic culture systems, focusing on gut microbiota composition, bile acid profiles, and related signaling pathways. These findings provide insight into the role of LCA in gut microbial ecology and host metabolic regulation.

## 2. Materials and Methods

### 2.1. Study Population

Data for this analysis were derived from our group’s previously published study in type 2 diabetes [13]. We reclassified participants by fasting plasma glucose (FPG) in accordance with ADA Standards of Care (T2DM: FPG ≥ 7.0 mmol/L, *n* = 80; controls: FPG < 7.0 mmol/L, *n* = 40) and compared serum bile acid profiles between groups.

### 2.2. Materials and Instruments

LCA (Sigma-Aldrich, St. Louis, MO, USA), metformin hydrochloride tablets (0.5 g per tablet; Merck Ltd., Darmstadt, Germany; batch ACD0048), and streptozotocin (MP Biomedicals, Solon, OH, USA; cat. no. 100557) were used in this study. Phosphate-buffered saline (PBS; Adamas Reagent Co., Shanghai, China; cat. no. C8020), Humulin insulin injection (Eli Lilly and Company, Indianapolis, IN, USA; batch D386919A), and citrate–sodium citrate buffer (SenBeiJia Biotechnology, Nanjing, China; cat. no. BL-S031) were used for sample preparation and physiological support.

The Concept 400 anaerobic workstation was sourced from Panmax Technology Co. (Garden City, NY, USA). The H1850 high-speed centrifuge was obtained from Xiang Yi Company (Jiaxing, China), and the Eppendorf 5424R refrigerated microcentrifuge was sourced from Eppendorf (Hamburg, Germany). The RTS-1C Automatic Microbial Growth Curve Analyzer was obtained from Guangzhou Shengrui Biotechnology Co. (Guangzhou, China).

### 2.3. Animal Study

Male C57BL/6 mice (7–8 weeks, 23–25 g) were acclimated for one week before random assignment into five groups: normal control (NC, *n* = 3), high-fat diet (HFD, *n* = 4), type 2 diabetes model (TM, *n* = 4), metformin-treated (MET, *n* = 4), and lithocholic acid-treated (LCA, *n* = 4). All animals were specific pathogen-free, wild-type mice and had not undergone any experimental procedures prior to study initiation. Animals were housed under a 12 h li12 h12-h dark cycle at room temperature (22–24 °C), with ad libitum access to food and water. No additional environmental enrichment was provided.

The experimental unit was a single animal. Animals were randomly allocated to experimental groups using a random number table. Potential confounders were minimized by maintaining identical conditions across groups, including temperature, humidity, light–dark cycle, diet, and handling procedures. No inclusion or exclusion criteria were defined a priori. All animals that entered the experiment were included in the analysis, and no animals or data points were excluded. These group sizes reflect the numbers of animals analyzed in each group. Group allocation was known to the investigator responsible for randomization, whereas outcome assessment and data analysis were performed blinded to group allocation.

Apart from NC, other groups were provided with a high-fat diet (D12492; Research Diets, lot 22090102), while NC received standard chow. MET and LCA groups received daily oral gavage of metformin (200 mg/kg) or lithocholic acid (50 mg/kg) for eight weeks; the remaining groups received saline. These procedures were designed to establish a T2DM model and to evaluate the metabolic effects of LCA. All animal procedures were approved by the Institutional Animal Care and Use Committee of the Shanghai Model Organisms Center (Approval Code: 2022-0044; Approval Date: 26 October 2022). A detailed animal use protocol, including the study objectives, experimental design, and planned procedures, was developed prior to study initiation and approved by the same IACUC.

Body weight was recorded weekly, and fasting glucose monitored biweekly. In week 6, TM, MET, and LCA mice were administered a single intraperitoneal dose of streptozotocin (40 mg/kg), whereas NC and HFD mice were given citrate buffer. Ten days after streptozotocin administration, metabolic evaluations were carried out, and body weight, fasting blood glucose, serum insulin concentrations, HOMA-IR, OGTT, and insulin sensitivity were selected as outcome variables.

Animals were routinely inspected throughout the study for overall health and well-being, with particular attention to body weight, food consumption, posture, spontaneous activity, grooming behavior, and coat appearance. All procedures were carried out in compliance with institutional animal welfare regulations, with measures taken to limit discomfort and distress. No adverse events, whether anticipated or unanticipated, occurred during the experimental period. Humane endpoints were not triggered, and no animals required premature removal from the study. This study was exploratory in design and did not define a single primary outcome for sample size determination.

Feces were collected 24 h prior to euthanasia and stored at −80 °C. At study endpoint, following a 12 h fast, mice were induced with 4% isoflurane anesthesia, and blood was drawn through cardiac puncture. After clotting at room temperature for 30 min, samples were centrifuged at 4 °C, and serum stored at −80 °C. Liver, spleen, and cecal contents were also harvested and frozen at −80 °C for subsequent analyses.

### 2.4. Biochemical Analyses

Fasting blood glucose was measured using an Accu Chek Performa meter (Roche Diagnostics, Basel, Switzerland) with compatible test strips. Fasting insulin (FINS) was measured using an insulin ELISA kit (Crystal Chem, Elk Grove Village, IL, USA, Cat. No. 90080). Assays for alanine aminotransferase (ALT), aspartate aminotransferase (AST), total cholesterol (TC), triglycerides (TG) were performed using kits from Nanjing Jiancheng Bioengineering Institute, Nanjing, China (Cat. Nos. C009-2-1, C010-2-1, A111-1-1, A110-1-1). The levels of lipopolysaccharide-binding protein (LBP) in mice were quantified using the Mouse LBP ELISA Kit (LPS Binding Protein) from Abcam, Cambridge, UK (Catalog No. ab269542).

### 2.5. OGTT and ITT Experiments

During the oral glucose tolerance test (OGTT), mice fasted for 6 h (ad libitum water). Baseline tail-tip blood was sampled at 0 min for FBG. A 50% glucose solution (2 g/kg) was administered by oral gavage, and additional tail-tip glucose measurements were taken at 15, 30-, 60-, 90-, and 120 min. Glucose tolerance was quantified by AUC analysis, and glucose responses were also analyzed after normalization to baseline.

During the insulin tolerance test (ITT), Following a 6 h fast, baseline blood glucose was recorded at 0 min, then insulin (0.5 U/kg) was given intraperitoneally. Blood glucose readings were collected at 15, 30-, 60-, 90-, and 120 min post-injection. Similarly, AUC values were derived from both raw glucose measurements and baseline-normalized responses in the ITT.

### 2.6. 16S rRNA Gene Sequencing

Fecal samples from each mouse group were collected, and genomic DNA was extracted using the OMEGA Soil DNA Kit (Omega Bio-Tek, Norcross, GA, USA). DNA quality was evaluated by Nanodrop spectrophotometry and electrophoresis on an agarose gel. The V3-V4 region of the 16S rRNA gene was amplified with universal primers, purified using Vazyme VAHTS™ DNA Clean Beads (Nanjing, China), and quantified with the PicoGreen assay. Amplicon sequencing was conducted on the Illumina NovaSeq 6000 platform, San Diego, CA, USA (2 × 250 bp). Raw sequences were demultiplexed with Demux, and primers were removed with Cutadapt. Quality control, denoising, and chimera removal were performed using DADA2 to create an amplicon sequence variant (ASV) table. Alpha and beta diversity were analyzed with QIIME2, and principal coordinate analysis (PCoA) was conducted. Taxa with differential abundance were identified using LEfSe (LDA score > 4).

### 2.7. Bile Acid Analysis

Bile acids in liver samples were analyzed using a Waters (Milford, MA, USA) ACQUITY UPLC HSS C18 column (1.8 μm, 100 mm × 2.1 mm). The mobile phase consisted of 0.01% acetic acid with 5 mmol/L ammonium acetate in ultrapure water (A) and 0.01% acetic acid in acetonitrile (B). Mass spectrometry was conducted in negative electrospray ionization (ESI) mode. Quantification was performed using multiple reaction monitoring (MRM) mode. Data acquisition and analysis were carried out with SCIEX Analyst 1.6.3 and MultiQuant 3.0.3 software. Standard curves were established using known standards, and integrated peak area ratios were applied to ensure accurate quantification.

### 2.8. In Vitro Impact of LCA on A. muciniphila Growth and Its Abundance in Fecal Microbiota

The *A. muciniphila* strain (ATCC BAA-835) used in this study was purchased from Beijing Baio Bio Biotechnology Co., Ltd. Prior to experimentation, the strain was activated in Brain Heart Infusion (BHI) medium (Hopebio, Qingdao, China) under anaerobic conditions at 37 °C for 24 h. After activation, the bacteria were exposed to different concentrations of LCA for 24 h. The range of LCA concentrations was selected based on previous studies that reported effective dose levels [14,15]. The optical density (OD) at 600 nm was measured every six hours to monitor bacterial growth.

A fecal sample was collected from an older individual who was clinically healthy and had not experienced any gastrointestinal conditions or been exposed to antibiotics or prebiotics in the preceding three months. The samples were suspended in a 0.1 mol/L phosphate-buffered saline solution (pH 7.2) at a mass-to-volume ratio of 10:1 and vortexed for two minutes to achieve homogenization. The mixture was centrifuged at 2000 rpm for 10 min. The supernatant and pellet were then collected separately. The supernatant was subjected to bile acid profiling, whereas the pellet served for 16S rRNA gene assessment and quantification of total bacterial load.

### 2.9. Quantification of Total Fecal Bacteria

Quantification of total bacterial abundance in sediment from in vitro fecal fermentation systems was achieved through real-time PCR. A plasmid harboring the full-length 16S rRNA gene derived from Lactobacillus served as the quantification reference. Serial dilutions of the plasmid, spanning from 10^3^ to 10^9^ copies per microliter, were prepared to construct standard curves. Amplification targeted the conserved bacterial 16S rRNA gene using a universal primer pair (Uni331F: 5′-TCCTACGGGAGGCAGCAGT-3′; Uni797R:5′-GGACTACCAGGGTATCTAATCCTGTT-3′), generating an amplicon of approximately 467 base pairs. Each PCR reaction was carried out in a final volume of 10 μL. Ct values obtained from the serial standards were plotted against log_10_-transformed copy numbers to generate the calibration curve. Absolute bacteria copy numbers in test samples were determined by substituting the corresponding Ct values into the regression model.

### 2.10. Measurement of GUS and BSH Enzyme Activity in Mouse Feces

Fecal samples (50 mg per mouse) were suspended in 0.1 M phosphate-buffered saline (PBS, pH 6.8), vortexed for 3 min, and homogenized. After centrifugation at 9000× *g* for 30 min at 4 °C, the supernatant was used to measure β-glucuronidase (β-GUS) activity. The assay mixture contained 20 μL supernatant, 175 μL PBS, and 5 μL of 4-methylumbelliferyl β-D-glucuronide (4-MUG), incubated at 37 °C for 30 min [16]. The reaction was stopped with acetonitrile, and fluorescence was measured at 340 nm excitation and 460 nm emission.

Fecal extracts were prepared as described for the GUS assay, and the clarified supernatant was used as the enzyme source. BSH activity was quantified with a far-red fluorogenic bile-acid substrate reported [17]. Briefly, the supernatant and probe were combined in PBS (pH 6.8) and incubated at 37 °C; fluorescence was recorded kinetically on a microplate reader. Heat-inactivated samples served as negative controls.

### 2.11. Real Time qPCR

Total RNA was carried out using the FastPure^®^ RNA Isolation Kit V2 (Vazyme, RC112). Reverse transcription was conducted with HiScript^®^ III RT SuperMix for qPCR (+gDNA wiper) (Vazyme, R323). Quantitative PCR (qPCR) was conducted using ChamQ SYBR qPCR Mix (Vazyme, Q711). Primers were available by Sangon Biotech (Shanghai) Co., Ltd. (Shanghai, China).

### 2.12. Statistical Analysis

In this study, statistical analyses were performed using GraphPad Prism 10.0 (GraphPad Software, San Diego, CA, USA). Comparisons were performed between the HFD and TM groups versus the NC group, and between the MET and LCA groups versus the TM group. For comparisons between two groups, unpaired or paired data was conducted using two-tailed Student’s *t*-test or the Mann–Whitney U test, depending on data distribution. For comparisons involving three or more groups, one-way analysis of variance (ANOVA) was employed, followed by Tukey’s multiple comparisons test. Nonparametric data were analyzed using the Kruskal–Walli’s test, with Dunn’s post hoc test for pairwise comparisons. Categorical data were assessed using Fisher’s exact test. In the figures, the “#” symbol indicates comparisons between the HFD or TM groups and the NC group, whereas the “*” symbol indicates comparisons between the Met or LCA groups and the TM group. All data are expressed as mean ± standard deviation (SD), and a *p*-value of less than 0.05 was considered statistically significant.

## 3. Results

### 3.1. Individuals with T2DM Exhibit Reduced LCA-Derived Bile Acid

**Figure 1:**
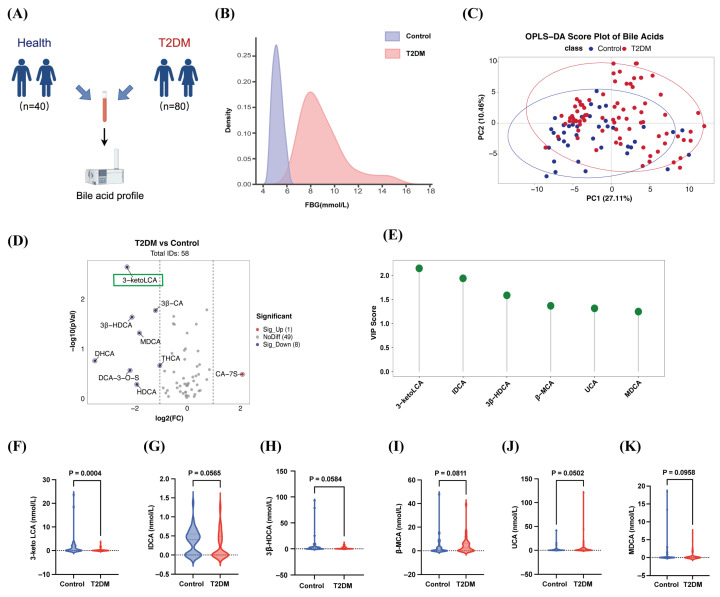
Individuals with T2DM exhibit reduced LCA-derived bile acid. (**A**) Plasma from 40 healthy and 80 T2DM individuals used for bile acid profiling. (**B**) FBG density curves in healthy vs. T2DM. (**C**) OPLS-DA shows separation of bile acid profiles by group. (**D**) Volcano plot showing differential bile acids by fold-change and significance. (**E**–**K**). Violin plots illustrate LCA-related bile acids presented according to VIP scores in the control and T2DM groups, including 3-keto LCA (**F**), IDCA (**G**), 3β-HDCA (**H**), β-MCA (**I**), UCA (**J**), and MDCA (**K**). Each point represents one subject. Group differences were assessed using the Mann–Whitney U test.

Building on our previous cohort [13], we compared the profile of serum bile acids in 40 healthy individuals and 80 patients with T2DM (Figure 1A,B). A supervised OPLS-DA model showed clear separation between the two groups based on the bile-acid matrix (Figure 1C). In the volcano plot, the T2DM group showed one bile acid significantly increased and eight significantly decreased relative to controls (Figure 1D). Applying *p* < 0.05 with VIP ≥ 1 retained six differential bile acids (Figure 1E–K); among them, 3-keto-LCA carried the top VIP (VIP value = 2.064), indicating the strongest driver of group separation.

3-keto-LCA, also known as 3-oxo-LCA, arises from LCA through C3-position oxidation as a downstream product [14]. Given the limited murine gavage literature on 3-keto-LCA and its unclear target profile, we administered its precursor, LCA, orally in mice to evaluate bile-acid-based intervention effects relevant to T2DM.

### 3.2. LCA Improves Glucose–Lipid Metabolism in T2DM Mice

**Figure 2:**
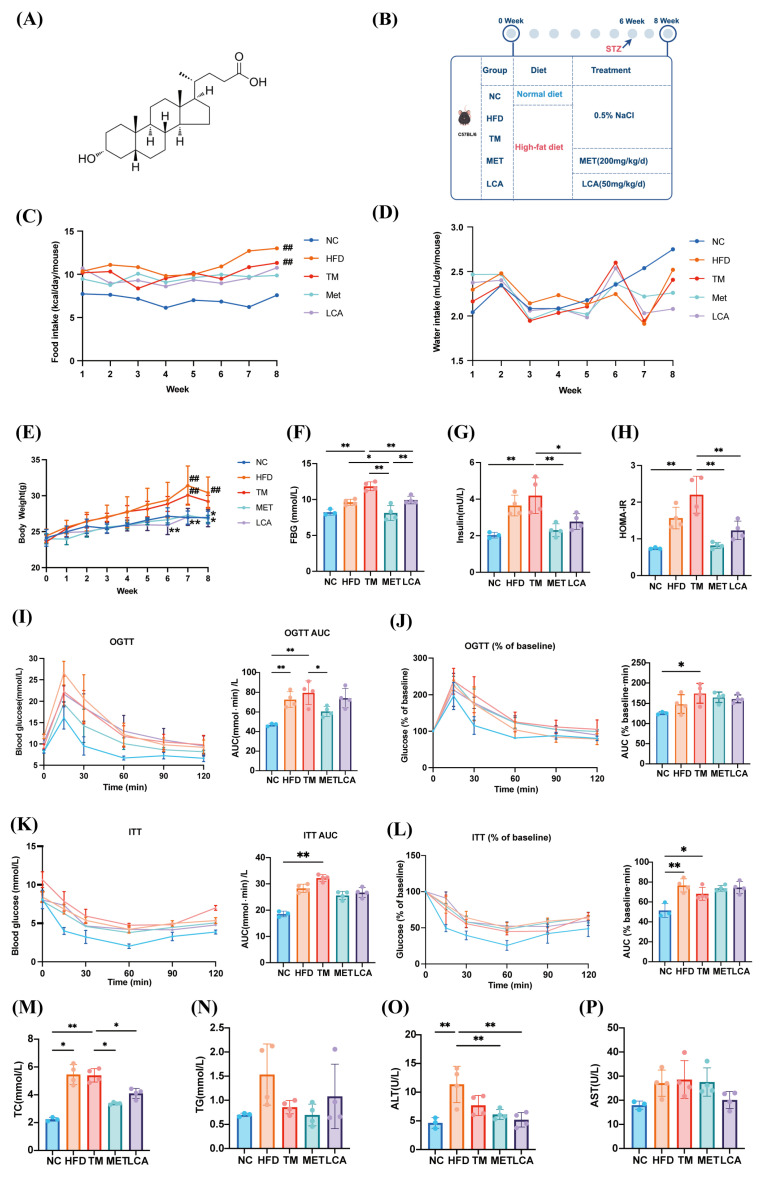
LCA Improves Glucose–Lipid Metabolism in T2DM Mice. (**A**) Molecular structure of LCA. (**B**) Schematic diagram of the experimental design and grouping strategy. (**C**,**D**) Weekly food intake and water intake. (**E**) Weekly body weight. (**F**) FBG levels. (**G**) Fasting insulin levels. (**H**) HOMA-IR index. (**I**,**J**) OGTT profiles including absolute glucose measurements at 0, 15, 30, 60, 90, and 120 min and AUC, as well as baseline-adjusted responses expressed as Gt/G0 × 100 with their respective AUC values. (**K**,**L**) ITT profiles showing absolute glucose responses at 0, 15, 30, 60, 90, and 120 min and AUC, together with baseline-adjusted glucose curves (Gt/G0 × 100) and AUC. (**M**) Serum TC level. (**N**) Serum TG level. (**O**) Serum ALT level. (**P**) Serum AST level. All data are presented as the mean ± SD. The “#” symbol indicates comparisons between the HFD or TM groups and the NC group (## *p* < 0.01), whereas the “*” symbol indicates comparisons between the Met or LCA groups and the TM group (* *p* < 0.05, ** *p* < 0.01).

To evaluate the metabolic benefits of LCA in T2DM, we employed a diet-and streptozotocin-induced mouse model of T2DM and administered LCA (50 mg/kg/day) for 4 weeks (Figure 2A,B). Food and water intake showed only minor fluctuations over the experimental period, with no marked differences among the HFD, TM, MET, and LCA groups, while the NC group consistently exhibited lower food intake and slightly higher water consumption (Figure 2C,D). Compared to TM group, LCA-treated mice significantly attenuated body weight gain throughout the intervention period (Figure 2E). LCA treatment significantly reduced both fasting blood glucose and insulin concentrations (Figure 2F,G), leading to a marked reduction in HOMA-IR values and indicating improved insulin sensitivity (Figure 2H). OGTT (Figure 2I,J) and ITT (Figure 2K,L) results not significantly different between the LCA and TM groups, although a smaller trend was observed.

In addition to regulating glucose, LCA has beneficial effects on the lipid profile and liver enzyme levels. LCA can significantly reduce TC, while TG levels show a downward trend, though this is not statistically significant (Figure 2K,L). Moreover, LCA attenuated liver injury, as indicated by reduced ALT activity and a downward trend in AST levels (Figure 2M,N).

Taken together, these data suggest that LCA provides T2DM mice with a range of metabolic benefits, including improved glucose and lipid metabolism, insulin sensitivity, and liver function.

### 3.3. LCA Reshapes Gut Microbial Composition and Enhances SCFA Production in T2DM Mice

**Figure 3:**
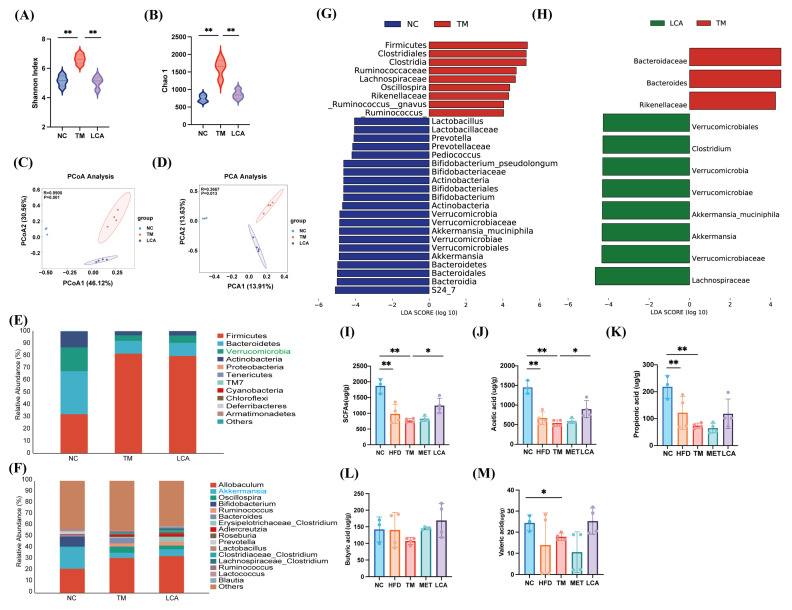
LCA intervention altered the gut microbiota composition and enhanced SCFA production in T2DM mice. (**A**) Shannon index. (**B**) Chao1 index. (**C**) Principal coordinate analysis (PCoA) based on Bray–Curtis distance. (**D**) Principal component analysis (PCA). (**E**) Relative abundance of gut microbiota at phylum level. (**F**) Relative abundance of gut microbiota at genus level. (**G**) LEfSe analysis identifying significantly different taxa between NC and TM groups (LDA > 4). (**H**) LEfSe analysis identifying significantly different taxa between TM and LCA groups (LDA > 4). (**I**) Total fecal SCFA levels. (**J**) Fecal acetate levels. (**K**) Fecal propionate levels. (**L**) Fecal butyrate levels. (**M**) Fecal valeric acid levels. All data are presented as the mean ± SD (* *p* < 0.05, ** *p* < 0.01).

This study employed 16S rRNA gene sequencing to investigate the impact of LCA on the gut microbiota. Alterations in the gut microbiota composition were observed in the T2DM mouse. Contrary to typical findings of reduced α-diversity in T2DM mice, the diabetic model in this study exhibited a significant increase in α-diversity. Following LCA treatment, Chao1 and Shannon index decreased significantly, approaching levels observed in the normal control group (Figure 3A,B). Further analysis of β-diversity based on PCoA and PCA revealed that the microbiota structure of LCA-treated mice was like that of the control group (Figure 3C,D).

Taxonomic profiling at phylum and genus levels revealed that the LCA significantly altered the relative abundance of major microbial taxa (Figure 3E,F). LEfSe analysis indicated that in TM group, dominant taxa included members of the phyla Firmicutes (e.g., Clostridiales, Ruminococcaceae) and Bacteroidetes (such as Bacteroides, Bacteroidaceae) (Figure 3G). In contrast, LCA treatment significantly enriched Verrucomicrobia, especially the genus *Akkermansia*, along with select Clostridium and Lachnospiraceae taxa relative to the TM group (Figure 3H). Interestingly, the taxon *Akkermansia*, along with its constituent species *A. muciniphila*, was also identified as characteristic of the NC group microbiota.

Moreover, targeted quantification of SCFAs revealed that total SCFAs, acetic acid, propionic acid and Valeric acid were significantly reduced in diabetic mice, whereas LCA supplementation markedly increased the levels of total SCFAs and acetate acid (Figure 3I–M). Although butyrate did not reach statistical significance, a rising trend was observed (Figure 3L). These results suggest that LCA mitigates T2DM-associated dysbiosis and restores beneficial microbial metabolites, which may contribute to improved host metabolic outcomes.

### 3.4. LCA Reshapes Hepatic Bile Acid Composition in T2DM Mice

**Figure 4:**
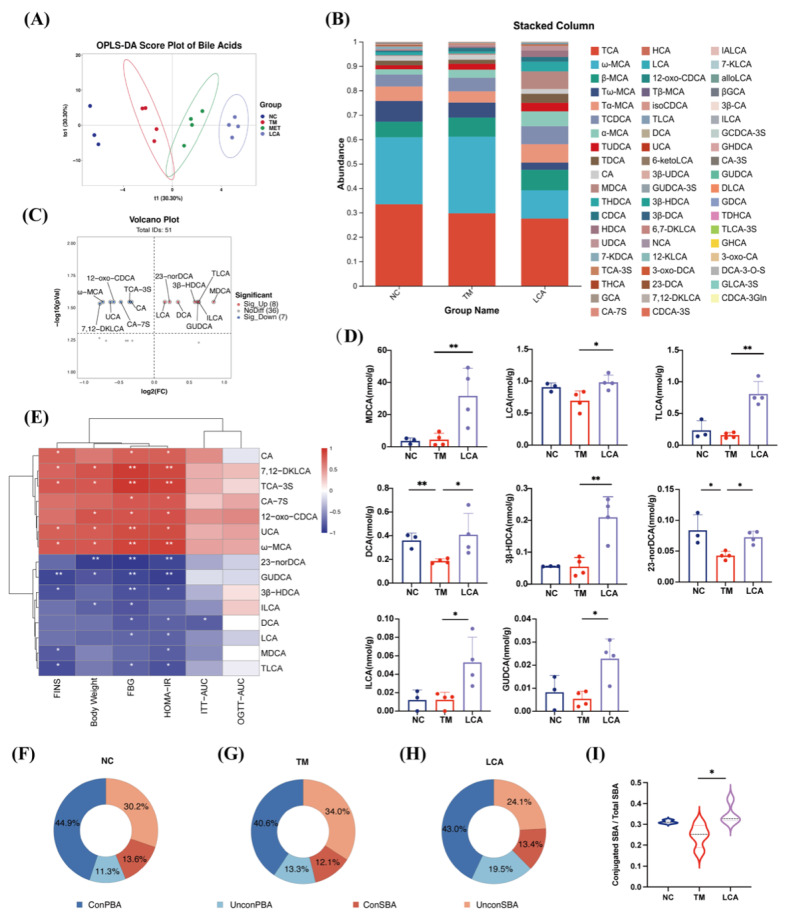
LCA reshapes hepatic bile acid composition in T2DM mice. (**A**) OPLS-DA score plot. (**B**) Stacked column chart. (**C**) Volcano plot of differential bile acids between TM and LCA groups. (**D**) Quantification of representative bile acids significantly elevated by LCA treatment. (**E**) Heatmap of correlations between bile acids and metabolic parameters. (**F**–**H**) Analysis of the Ratio of Conjugated to Unconjugated Bile Acids in the NC (**F**), TM (**G**), and LCA (**H**) Groups. (**I**) Ratio of conjugated to total secondary bile acids. All data are presented as the mean ± SD (* *p* < 0.05, ** *p* < 0.01).

To assess the effect of LCA on bile acid metabolism in diabetic mice, hepatic bile acid profiling was performed. OPLS-DA plots showed a clear separation between NC, TM, and LCA groups, while stacking plots showed changes in multiple bile acid levels after LCA treatment (Figure 4A,B). Eight bile acids were elevated, and seven other bile acids were decreased in LCA-treated mice compared with the TM group. Notably, multiple bile acids exhibited significant alterations following exposure to LCA. Elevated species included murine deoxycholic acid (MDCA), LCA and glycyrlurea deoxycholic acid (GUDCA). In contrast, significant reductions in 12-oxo chenodeoxycholic acid (12 oxo CDCA) and ωMouse cholic acid (ωMCA), as well as several other bile acid derivatives, were observed (Figure 4C,D). Spearman’s analysis of blood glucose indices and differential bile acids revealed a negative correlation between elevated bile acids, including LCA, MDCA and GUDCA and blood glucose indices such as FBG and HOMA-IR. This suggests that these bile acids may play a role in maintaining blood glucose homeostasis (Figure 4E).

Further classification of the bile acid profile showed that treatment with LCA increased the proportion of conjugated species, especially secondary conjugated bile acids (Figure 4F–H). Conjugated secondary bile acid levels were significantly increased in the LCA group (Figure 4I), indicating changes in bile acid metabolism and conjugation patterns. Taken together, these findings suggest that LCA’s improvement in glycemic control may be related to changes in the composition and structure of bile acids.

### 3.5. LCA Strengthens the Gut Barrier and Reduces Fecal GUS and BSH Activities

**Figure 5:**
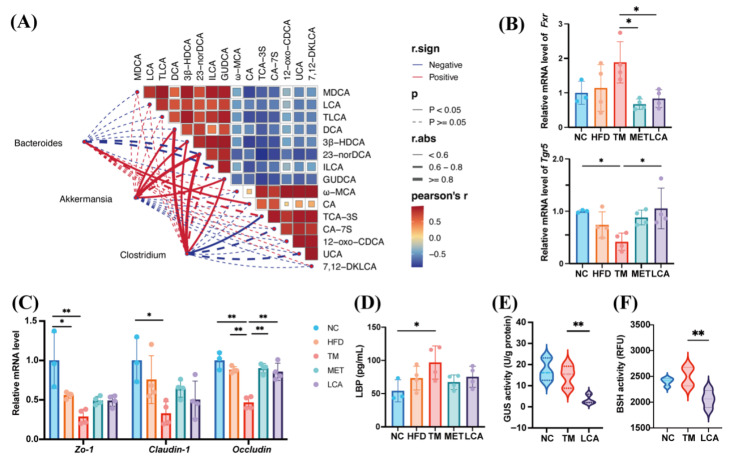
LCA Strengthens the Gut Barrier and Reduces Fecal GUS and BSH Activities. (**A**) Tripartite correlation network showing associations among gut microbiota, bile acids, and key metabolic parameters based on Spearman’s correlation analysis. (**B**) mRNA expression of *Fxr* and *Tgr5* in ileal tissue. (**C**) Relative mRNA levels of tight junction proteins *Zo-1*, *Claudin-1*, and *Occludin* in the ileum. (**D**) Serum LBP concentration. (**E**) Fecal GUS activity. (**F**) Fecal BSH activity. All data are presented as the mean ± SD (* *p* < 0.05, ** *p* < 0.01).

To investigate the relationship between gut microbiota and bile acid metabolism in T2DM mice, we first performed LEfSe analysis to identify differentially abundant taxa at the genus and species levels between the TM and LCA groups (Figure 3H). Subsequently, based on the differential bile acid profiles between the two groups (Figure 4C), Spearman’s rank correlation analysis was conducted to assess the associations between these differentially abundant taxa and bile acid metabolites (Figure 5A). Significant positive or negative correlations were observed between bacterial genera such as Bacteroides, *Akkermansia* and *Clostridium*, and bile acid metabolites such as DCA, MDCA and LCA. Notably, *Akkermansia* exhibited a strong positive correlation with several bile acids, particularly DCA, 3β-HDCA, 23-norDCA, and GUDCA (Spearman’s ρ > 0.6, *p* < 0.05).

The regulatory influence of LCA on bile acid receptors was investigated by measuring the levels of Tgr5 and Fxr mRNA in the ileal tissue of mice. Compared to the TM group, Tgr5 expression increased significantly, while Fxr expression decreased notably in the LCA-treated group (Figure 5B). Moreover, LCA could significantly increase the level of *occludin* at the gene level (Figure 5C). Lipopolysaccharide binding protein (LBP) levels were significantly decreased in TM group and decreased after LCA treatment, which was close to the control level (Figure 5D). Furthermore, the activities of GUS and BSH enzymes were significantly lower in the feces of LCA group mice than in those of the model group (Figure 5E,F).

Overall, LCA’s improvement of metabolic homeostasis may be related to modulation of the intestinal microbe-bile acid network, as well as *Tgr5* activation and *Fxr* inhibition.

### 3.6. LCA Enhances the Growth of A. muciniphila and Reshapes Fecal Bile Acid Profiles In Vitro

**Figure 6:**
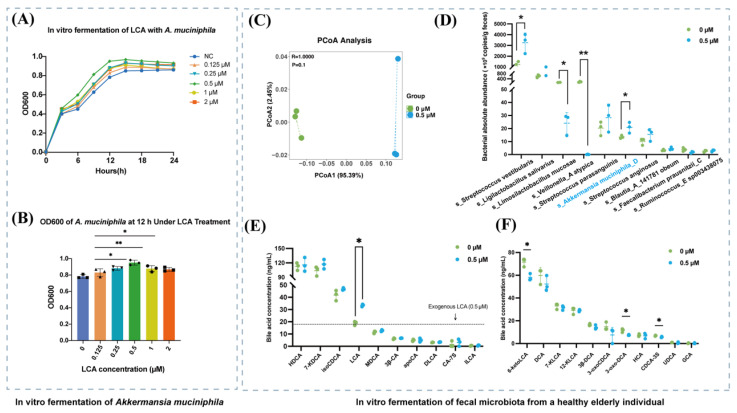
LCA enhances *A. muciniphila* growth and alters fecal bile acid profiles in vitro. (**A**) Growth curves of *A. muciniphila* treated with increasing LCA concentrations, measured by OD600. (**B**) OD600 values at 12 h from (**A**). (**C**) PCoA based on Bray–Curtis distances of in vitro fecal samples treated with or without 0.5 μM LCA. (**D**) Estimated absolute abundance of the ten most dominant bacterial taxa in fecal culture samples subjected to 0 or 0.5 μM LCA, calculated using total bacterial copy number combined with 16S rRNA gene profiling. (**E**,**F**) Bile acid levels in fermentation supernatants following 0.5 μM LCA treatment. All data are presented as the mean ± SD (* *p* < 0.05, ** *p* < 0.01).

To assess the direct growth-promoting effect of LCA on *A. muciniphila*, gradient concentrations of LCA were co-cultured with Akk, and OD600 was measured every 6 h. The results indicated that LCA treatment caused an initial increase in Akk growth followed by stabilization, with the peak OD600 reached at 12 h for most concentrations (Figure 6A). Therefore, 12 h was selected as the optimal time point for analysis. At this time point, 0.5 μM LCA was determined to be the most effective concentration for promoting Akk growth (Figure 6B).

To investigate the influence of LCA on *A. muciniphila* within a complex microbial environment, an in vitro fermentation model was constructed using fecal samples from a healthy elderly donor. PCoA analysis revealed that the 0.5 μM LCA treatment group and the untreated group were partially distinct on PCoA1, which accounted for 95.39% of the variation. This suggests that this concentration of LCA could alter the composition of microbes (Figure 6C). Treatment with 0.5 μM LCA significantly altered the abundance of specific bacteria, increasing the abundance of *A. muciniphila* (*p* = 0.042) and *S. vestibularis* (*p* = 0.021). Conversely, *Limosilactobacillus mucosae* (*p* = 0.026) and *Veillonella atypica* (*p* = 0.002) decreased in abundance. These results show that LCA can selectively promote the growth of certain species, such as *A. muciniphila*, thereby reshaping the complex gut microbiota (Figure 6D). Analysis of bile acid targeting showed that exposure to 0.5 μM LCA significantly altered the composition of bile acids, resulting in a notable decrease in oxygen-derived bile acids, including 3α-Hydroxy-6-oxo-5β-cholan-24-oic acid (6-ketoLCA), 3-oxochenodeoxycholic acid (3-oxoDCA) and Chenodeoxycholic acid 3-sulfate disodium salt (CDCA-3S) (Figure 6E,F). These results suggest that LCA increases the abundance of *A. muciniphila* in both pure cultures and complex communities, while also significantly altering the bile acid profile in vitro.

## 4. Discussion

Bile acids are closely linked with glucose metabolism. In our human cohort, patients with T2D exhibit a significant reduction in LCA-related downstream metabolites versus healthy individuals. LCA gavage enhanced glucose homeostasis in a T2D mouse model. Mechanically, LCA exposure increased the abundance of *A. muciniphila* in vivo and in vitro and decreased fecal BSH and GUS activities. LCA also shifted the hepatic bile acid pool in T2D mice towards a healthier metabolic state. Overall, LCA appears to enhance glycemic control by remodeling the gut microbiome and bile-acid axis in a coordinated way.

Although in low abundance, it plays an important role in mediating signals in metabolic diseases [18]. Studies have shown that metabolites derived from LCA can inhibit Th17 differentiation and promote the induction of Foxp3^+^ Tregs, thereby helping to maintain intestinal immune homeostasis [19,20]. In animal models, circulating levels of LCA were found to be negatively correlated with OGTT AUC [21]. In our human cohort, levels of 3-keto LCA, a downstream metabolite of LCA, were significantly lower in patients with T2DM. Then we conducted an oral intervention using LCA in a mouse model and discovered that it effectively reduced fasting glucose, insulin, HOMA-IR and TG levels. This suggests that LCA can improve overall glucose and lipid metabolic homeostasis.

Based on this, we explored the potential mechanisms by which LCA improves metabolic disorders. The 16S rRNA gene sequencing revealed that LCA altered the composition of the gut microbiota and increased the level of *A. muciniphila* in mice with T2DM. To confirm whether LCA directly drives microbial changes, single-strain culture was performed, revealing that LCA could significantly promote the growth of *A. muciniphila* at low concentrations. Furthermore, LCA was shown to consistently increase the abundance of fecal microbiota in in vitro community cultures of healthy older adults. Overall, LCA is shown to consistently increase the abundance of *A. muciniphila* in both in vivo and in vitro studies, suggesting that its metabolic benefits may be closely associated with the enrichment of this key bacterium.

*A. muciniphila* is a new generation of probiotics with multiple beneficial effects on host metabolism [22]. Acetate and other short-chain fatty acids are produced in large quantities during mucin fermentation by this species [23], and accordingly acetate levels were significantly elevated in our LCA-treated group. These SCFAs can enhance insulin sensitivity and reduce inflammation by acting in various ways [24,25,26]. *A. muciniphila* can also reduce endotoxin translocation and improves intestinal barrier function [27,28]. The enrichment of *A. muciniphila* in LCA-treated T2DM mice was accompanied by an increase in *Occludin* transcripts and a decrease in serum LBP, indicating improved intestinal barrier function. Taken together with our in vitro and in vivo results, LCA acts as an internal regulator that improves metabolic health by modulating the ecology of gut microbes.

Gut bacterial enzymes reshape bile acid composition to support metabolic homeostasis [29]. The deglucuronidation of conjugated compounds is driven by microbiota-derived GUS [30], and reducing GUS activity increases the removal of polar bile acids and reduces epithelial stress [31]. BSH hydrolyze bound bile salts to form free bile acids, which in turn alter the ligands of bile acid receptors FXR and TGR5, affecting metabolic signaling [32,33]. In our study, the group treated with LCA had reduced fecal GUS and BSH activities, leading to changes in the bile acid pool and improvement of glucose metabolism.

Our results suggest that LCA alters the profile of bile acids in both in vivo and in vitro. We observed changes in the levels of several bile acids, particularly ILCA and GUDCA, in mice treated with LCA. Higher levels of ILCA in the blood were linked to a lower risk of type 2 diabetes and a lower BMI in large group studies [34]. LCA can inhibit the differentiation of Th17 cells and enhance intestinal immunity by antagonizing RORγt [35]. Another key metabolite, GUDCA, acts as an FXR antagonist, alleviating ER stress and improving insulin sensitivity [36,37,38]. Consistently, we observed a decrease in intestinal FXR and an increase in TGR5 mRNA, which is known to regulate blood glucose levels by promoting GLP-1 secretion [39]. In our LCA-treated mice, levels of LCA, ILCA and GUDCA were positively correlated with the abundance of *A. muciniphila*, particularly GUDCA. There was also a negative correlation with FBG and HOMA-IR. This highlights the contribution of these bile acids to glycemic improvement in vivo.

Whether the observations reported here extend to other species or are applicable to human metabolic disorders remains uncertain. Nevertheless, the pathways examined are central to metabolic regulation and provide a context for subsequent studies in human-relevant systems. A limitation of the present study is that the observed metabolic effects of LCA, although consistent with modulation of the gut microbiota–bile acid axis, were evaluated exclusively in experimental models. Confirmation of these findings in human-relevant systems, including intestinal organoids or clinical settings, remains to be addressed in future work.

In conclusion, we observed significant differences in bile acid profiles when comparing healthy individuals with T2DM patients. The effects of LCA on glucose and lipid metabolism were then validated in a mouse model. The underlying mechanism appears to involve the selective enrichment of *Akkermansia*, the downregulation of GUS and BSH enzyme activities in the gut and the reprogramming of the bile acid profile. Our findings suggest that LCA derived from digestive processes may represent a novel dietary nutritional intervention strategy for managing type 2 diabetes by modulating the microbiota.

## 5. Conclusions

By integrating data from human cohorts, animal models and in vitro experiments, this research shows that LCA acts in a cooperative way to regulate the gut microbiota-bile acid-host signaling axis, thereby improving glucose and lipid metabolism. LCA metabolite levels were significantly lower in T2DM patients. In T2DM mice, the glucose and lipid metabolism indexes improved by the administration of LCA. Mechanistically, these benefits are closely linked to LCA-driven gut microbiota remodeling and alterations to bile acid profiles. LCA reduced BSH and GUS activities, increased SCFA levels, and enhanced intestinal barrier integrity in T2DM mice. Notably, LCA significantly increased the abundance of *A. muciniphila* in both in vivo and in vitro, while remodeling the bile acid pool. These findings reveal the metabolic benefits of LCA, suggesting its potential as a functional component in the development of functional foods or probiotic preparations for improving glycolipid health.

## Data Availability

Raw 16S rRNA gene sequencing reads have been deposited in the Genome Sequence Archive (GSA) at the National Genomics Data Center, China National Center for Bioinformation (CNCB) under accession CRA032038.

## Abbreviations

The following abbreviations are used in this manuscript:

T2DM	Type 2 Diabetes Mellitus
GC–MS	Gas Chromatography–Mass Spectrometry
LCA	Lithocholic Acid
HOMA-IR	Homeostatic Model Assessment of Insulin Resistance
16S rRNA	16S Ribosomal RNA
SCFAs	Short-Chain Fatty Acids
BSH	Bile Salt Hydrolase
TGR5	Takeda G-Protein-Coupled Receptor 5
FXR	Farnesoid X Receptor
Akk	Akkermansia muciniphila
BAs	Bile Acids
GLP-1	Glucagon-Like Peptide 1
TβMCA	Tauro-β-Muricholic Acid
FPG	Fasting Plasma Glucose
PBS	Phosphate-Buffered Saline
BHI	Brain Heart Infusion
NC	Normal Control
HFD	High-Fat Diet
TM	Type 2 Diabetes Model
MET	Metformin
FBG	Fasting Blood Glucose
FINS	Fasting Insulin
ALT	Alanine Aminotransferase
AST	Aspartate Aminotransferase
TC	Total Cholesterol
TG	Triglyceride
LBP	Lipopolysaccharide-Binding Protein
OGTT	Oral Glucose Tolerance Test
AUC	Area Under the Curve
ITT	Insulin Tolerance Test
PCoA	Principal Coordinate Analysis
PCA	Principal Component Analysis
LEfSe	Linear Discriminant Analysis Effect Size
MDCA	Murichodeoxycholic Acid
GUDCA	Glycoursodeoxycholic Acid
SCFA	Short-Chain Fatty Acid
ASV	Amplicon Sequence Variant
UPLC	Ultra-Performance Liquid Chromatography
ESI	Electrospray Ionization
MRM	Multiple Reaction Monitoring
OD600	Optical Density at 600 nm
Ct	Cycle Threshold
PCR	Polymerase Chain Reaction
qPCR	Quantitative Polymerase Chain Reaction
GUS	β-Glucuronidase
6-ketoLCA	3α-Hydroxy-6-oxo-5β-cholan-24-oic Acid
3-oxoDCA	3-Oxo-Deoxycholic Acid
CDCA-3S	Chenodeoxycholic Acid-3-Sulfate
ILCA	Iso-Lithocholic Acid
RORγt	Retinoic-Acid-Related Orphan Receptor Gamma t
BMI	Body Mass Index
GSA	Genome Sequence Archive
CNCB	China National Center for Bioinformation
